# Cost-Sharing of Ecological Construction Based on Trapezoidal Intuitionistic Fuzzy Cooperative Games

**DOI:** 10.3390/ijerph13111102

**Published:** 2016-11-08

**Authors:** Jiacai Liu, Wenjian Zhao

**Affiliations:** 1 College of Transportation and Civil Engineering, Fujian Agriculture and Forestry University, Fuzhou 350002, China; 2 Jinshan College, Fujian Agriculture and Forestry University, Fuzhou 350002, China; zhaowenjian66@126.com

**Keywords:** cost-sharing, ecological construction, fuzzy games, cooperative games, trapezoidal intuitionistic fuzzy numbers

## Abstract

There exist some fuzziness and uncertainty in the process of ecological construction. The aim of this paper is to develop a direct and an effective simplified method for obtaining the cost-sharing scheme when some interested parties form a cooperative coalition to improve the ecological environment of Min River together. Firstly, we propose the solution concept of the least square prenucleolus of cooperative games with coalition values expressed by trapezoidal intuitionistic fuzzy numbers. Then, based on the square of the distance in the numerical value between two trapezoidal intuitionistic fuzzy numbers, we establish a corresponding quadratic programming model to obtain the least square prenucleolus, which can effectively avoid the information distortion and uncertainty enlargement brought about by the subtraction of trapezoidal intuitionistic fuzzy numbers. Finally, we give a numerical example about the cost-sharing of ecological construction in Fujian Province in China to show the validity, applicability, and advantages of the proposed model and method.

## 1. Introduction

Owing to the fuzziness and uncertainty in some decision problems, fuzzy cooperative games have been extensively studied and successfully applied to many areas such as the economy, management, ecology, politics, and diplomacy [1,2,3]. In fact, sometimes it is quite difficult for players to estimate exactly the coalition values once they form a coalition to do something together. Generally, we can use the theory and method of fuzzy games [4,5,6,7], interval games [8,9,10,11], and stochastic games [12] to deal with the lack of precision and the distortion of information.

Intuitionistic fuzzy cooperative games play a very important role in situations involving imprecise and inadequate information in the process of decision making. The complete ranking of intuitionistic fuzzy numbers is an open problem all over the world. Researchers worldwide have been studying the ranking of several types of intuitionistic fuzzy numbers since 1985, and several ranking methods have been proposed to date. Lakshmana et al. [13] introduced a linear (total) ordering on the class of trapezoidal intuitionistic fuzzy numbers using an axiomatic set of eight different scores. Nehi [14] proposed a new ordering method for intuitionistic fuzzy numbers and made some operations on them. Garg [15] presented an alternative method to construct the membership function under an intuitionistic fuzzy environment. Wan [16] developed some new generalized aggregation operators for triangular intuitionistic fuzzy numbers and applied them to multi-attribute group decision making problems. Li [17] proposed a new ranking method based on the concept of a ratio of the value index to the ambiguity index and applied the new method to multi-attribute decision making problems. However, there is nearly no solving method of the membership, non-membership, and hesitance degree considering the effect of the numerical value part of intuitionistic fuzzy numbers.

There also exist fuzziness and uncertainty in the process of ecological construction, so it is difficult to obtain the cost-sharing scheme according to classical cooperative games. Ecological construction has attracted the attention of many researchers. For the past few years, the research on ecological construction has focused on the problem and countermeasure of ecological construction [18], the relationship between ecological construction and economic development [19,20], the interaction between ecological construction and environmental protection [21,22], and the allocation method of ecological compensation [23,24,25,26,27,28]. Through looking up the existing literature, the conclusion can be drawn that there has been little study on the cost-sharing of ecological construction, especially based on the theory and method of fuzzy cooperative games. However, in the process of ecological construction, because of the long cycle of construction, the substantial input of funds, and the fuzziness and uncertainty existing in the process of decision making, it is almost impossible to precisely estimate the construction cost. Under these circumstances, it is more suitable for us to solve the problem of the cost-sharing of ecological construction using fuzzy cooperative games.

The rest of this paper is organized as follows. Section 2 gives the definition of trapezoidal intuitionistic fuzzy numbers and briefly reviews some mainly arithmetical operations of trapezoidal intuitionistic fuzzy numbers. In Section 3, based on the square of the distance in the numerical value between two trapezoidal intuitionistic fuzzy numbers and the least square method, we construct two quadratic programming models to solve cooperative games with trapezoidal intuitionistic fuzzy numbers. In Section 4, an example about the cost-sharing of ecological construction in Fujian province, China is given to show the validity, applicability, and advantages of the proposed model and method. Section 5 makes some discussions about the model and method proposed in this paper. The paper’s conclusion is provided in Section 6.

## 2. Definition and Arithmetical Operations for Trapezoidal Intuitionistic Fuzzy Numbers

### 2.1. Definition of Trapezoidal Intuitionistic Fuzzy Numbers

**Definition** **1.** *Let $\tilde{a}=<(a,b_{1},b_{2},c);\;w_{\tilde{a}},u_{\tilde{a}}>$, where $a\leq b_{1}\leq b_{2}\leq c$, be a special intuitionistic fuzzy based on the set of real numbers. If the membership function and nonmembership function are defined as follows [29]:*

(1) $$\mu_{\tilde{a}}(x)=\left\{ \begin{array}{ll} (x-a)w_{\tilde{a}}/(b_{1}-a) & \;(a\leq x<b_{1})\\ w_{\tilde{a}} & \left(b_{1}\leq x\leq b_{2}\right)\\ (c-x)w_{\tilde{a}}/(c-b_{2}) & \;(b_{2}<x\leq c)\\ 0 & \left(x<a,x>c\right) \end{array}\right.$$

*and*

(2) $$\upsilon_{\tilde{a}}(x)=\left\{ \begin{array}{ll} \left[b_{1}-x+u_{\tilde{a}}(x-a)\right]/(b_{1}-a) & \;(a\leq x<b_{1})\\ u_{\tilde{a}} & \left(b_{1}\leq x\leq b_{2}\right)\\ \left[x-b_{2}+u_{\tilde{a}}(c-x)\right]/(c-b_{2}) & \;(b_{2}<x\leq c)\\ 1 & (x<a,x>c), \end{array}\right.$$

**Figure 1:**
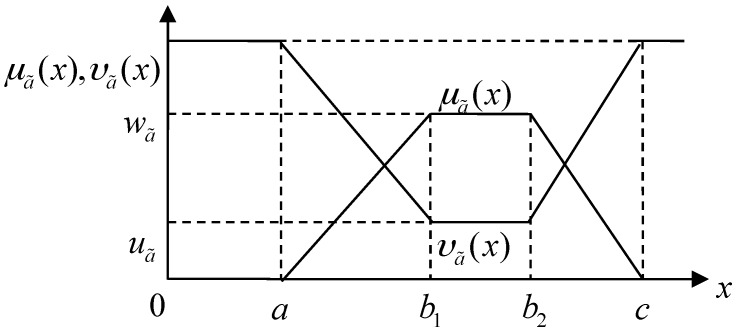
A trapezoidal intuitionistic fuzzy number $\tilde{a}=<(a,b_{1},b_{2},c);w_{\tilde{a}},u_{\tilde{a}}>$.

*respectively, where a, $b_{1}$, $b_{2}$, and c are all real numbers and the values $w_{\tilde{a}}$ and $u_{\tilde{a}}$ satisfy the following conditions: $0\leq w_{\tilde{a}}\leq 1$, $0\leq u_{\tilde{a}}\leq 1$, and $0\leq w_{\tilde{a}}+u_{\tilde{a}}\leq 1$, then the fuzzy number $\tilde{a}=<(a,b_{1},b_{2},c);\;w_{\tilde{a}},u_{\tilde{a}}>$ is called the trapezoidal intuitionistic fuzzy number, depicted as in Figure 1*.

In an arbitrary trapezoidal intuitionistic fuzzy number $\tilde{a}=<(a,b_{1},b_{2},c);w_{\tilde{a}},u_{\tilde{a}}>$, $w_{\tilde{a}}$ and $u_{\tilde{a}}$ represent the maximum degree of membership and the minimum degree of nonmembership, respectively. Let $\pi_{\tilde{a}}(x)=1-\mu_{\tilde{a}}(x)-\upsilon_{\tilde{a}}(x)$, then the function $\pi_{\tilde{a}}(x)$, which is called the degree of hesitancy, denotes the hesitation of an element x in the trapezoidal intuitionistic fuzzy number $\tilde{a}$. It is obvious that the smaller $\pi_{\tilde{a}}(x)$ is, the more certain $\tilde{a}$ becomes.

If a≥0 and the other three values $b_{1}$, $b_{2}$, and c are simultaneously not equal to 0, then the trapezoidal intuitionistic fuzzy number $\tilde{a}=<(a,b_{1},b_{2},c);w_{\tilde{a}},u_{\tilde{a}}>$ is called a positive trapezoidal intuitionistic fuzzy number, denoted by $\tilde{a}>0$. Similarly, if c≤0 and at least one of the other three values $b_{1}$, $b_{2}$, and c is less than 0, than the trapezoidal intuitionistic fuzzy number $\tilde{a}=<(a,b_{1},b_{2},c);w_{\tilde{a}},u_{\tilde{a}}>$ is called a negative trapezoidal intuitionistic fuzzy number, denoted by $\tilde{a}<0$. In the following sections, we only discuss the positive trapezoidal intuitionistic fuzzy number.

A trapezoidal intuitionistic fuzzy number $\tilde{a}=<(a,b_{1},b_{2},c);w_{\tilde{a}},u_{\tilde{a}}>$ can express fuzziness and uncertainty more felicitously than a triangular intuitionistic fuzzy number ${\tilde{a}}^{\prime }=<(a,b,c);w_{\tilde{a}},u_{\tilde{a}}>$ or a trapezoidal fuzzy number ${\tilde{a}}^{\prime \prime }=(a,b_{1},b_{2},c)$. However, the three types of fuzzy numbers mentioned above have inextricable connections. Particularly, a triangular intuitionistic fuzzy number ${\tilde{a}}^{\prime }=<(a,b,c);w_{\tilde{a}},u_{\tilde{a}}>$ is the special case of a trapezoidal intuitionistic fuzzy number $\tilde{a}=<(a,b_{1},b_{2},c);w_{\tilde{a}},u_{\tilde{a}}>$ when $b_{1}=b_{2}$, and a trapezoidal fuzzy number ${\tilde{a}}^{\prime \prime }=(a,b_{1},b_{2},c)$ is the special case of a trapezoidal intuitionistic fuzzy number $\tilde{a}=<(a,b_{1},b_{2},c);w_{\tilde{a}},u_{\tilde{a}}>$ when $w_{\tilde{a}}=1$ and $u_{\tilde{a}}=0$. In other words, if $b_{1}=b_{2}$, a trapezoidal intuitionistic fuzzy number $\tilde{a}=<(a,b_{1},b_{2},c);w_{\tilde{a}},u_{\tilde{a}}>$ reduces to a triangular intuitionistic fuzzy number ${\tilde{a}}^{\prime }=<(a,b,c);w_{\tilde{a}},u_{\tilde{a}}>$. Moreover, if $w_{\tilde{a}}=1$ and $u_{\tilde{a}}=0$, a trapezoidal intuitionistic fuzzy number $\tilde{a}=<(a,b_{1},b_{2},c);w_{\tilde{a}},u_{\tilde{a}}>$ reduces to a trapezoidal fuzzy number ${\tilde{a}}^{\prime \prime }=(a,b_{1},b_{2},c)$.

A trapezoidal intuitionistic fuzzy number $\tilde{a}=<(a,b_{1},b_{2},c);w_{\tilde{a}},u_{\tilde{a}}>$ may represent approximately a fuzzy value between $b_{1}$ and $b_{2}$. Any intermediate value $x\in [b_{1},b_{2}]$ has the same probability to become the most possible value with the membership degree $w_{\tilde{a}}$ and nonmembership degree $u_{\tilde{a}}$. The most impossible values are a and c, which are called the pessimistic value and the optimistic value, respectively. Both of the two most impossible values have the same membership degree 0 and nonmembership degree 1. For any other value x∈[a,c], the membership degree is obtained by $\mu_{\tilde{a}}(x)$ and the nonmembership degree is obtained by $\upsilon_{\tilde{a}}(x)$. The range of possible values of the trapezoidal intuitionistic fuzzy number $\tilde{a}=<(a,b_{1},b_{2},c);w_{\tilde{a}},u_{\tilde{a}}>$ is determined by a and c, which are called the lower and upper limits of the trapezoidal intuitionistic fuzzy number $\tilde{a}=<(a,b_{1},b_{2},c);w_{\tilde{a}},u_{\tilde{a}}>$, respectively.

### 2.2. Arithmetical Operationsfor Positive Trapezoidal Intuitionistic Fuzzy Numbers

Owing to the fact that it is meaningless if the coalition values or payoffs of players are negative, in this paper, we do not discuss the negative trapezoidal intuitionistic fuzzy number. Therefore, we only give some common operation laws for positive trapezoidal intuitionistic fuzzy numbers in this section.

**Definition** **2.** *Let ${\tilde{a}}_{1}=<(a_{1},b_{11},b_{21},c_{1});w_{{\tilde{a}}_{1}},u_{{\tilde{a}}_{1}}>$ and ${\tilde{a}}_{2}=<(a_{2},b_{12},b_{22},c_{2});w_{{\tilde{a}}_{2}},u_{{\tilde{a}}_{2}}>$ be two arbitrary positive trapezoidal intuitionistic fuzzy numbers and λ≠0 be any real number. Then, the main algebraic operations are stipulated as follows [29,30]:*

(3) $${\tilde{a}}_{1}+{\tilde{a}}_{2}=<(a_{1}+a_{2},b_{11}+b_{12},b_{21}+b_{22},c_{1}+c_{2});w_{{\tilde{a}}_{1}}\wedge w_{{\tilde{a}}_{2}},u_{{\tilde{a}}_{1}}\vee u_{{\tilde{a}}_{2}}>$$

(4) $${\tilde{a}}_{1}-{\tilde{a}}_{2}=<(a_{1}-c_{2},b_{11}-b_{22},b_{21}-b_{12},c_{1}-a_{2});w_{{\tilde{a}}_{1}}\wedge w_{{\tilde{a}}_{2}},u_{{\tilde{a}}_{1}}\vee u_{{\tilde{a}}_{2}}>$$

(5) $${\tilde{a}}_{1}\times {\tilde{a}}_{2}=<(a_{1}a_{2},b_{11}b_{12},b_{21}b_{22},c_{1}c_{2});w_{{\tilde{a}}_{1}}\wedge w_{{\tilde{a}}_{2}},u_{{\tilde{a}}_{1}}\vee u_{{\tilde{a}}_{2}}>$$

*and*

(6) $$\lambda {\tilde{a}}_{1}=\left\{ \begin{array}{ll} <(\lambda a_{1},\lambda b_{11},\lambda b_{21},\lambda c_{1});w_{{\tilde{a}}_{1}},u_{{\tilde{a}}_{1}}> & \left(\lambda >0\right)\\ <(\lambda c_{1},\lambda b_{21},\lambda b_{11},\lambda a_{1});w_{{\tilde{a}}_{1}},u_{{\tilde{a}}_{1}}> & \left(\lambda <0\right) \end{array}\right.$$

*where the symbols “∧” and “∨” mean minimum and maximum operators, respectively.*

According to the main algebraic operations of trapezoidal intuitionistic fuzzy numbers (i.e., Equations (3)–(6)), conclusions can be easily drawn that the forward part (i.e., $\left(a,b_{1},b_{2},c\right)$) and the latter part (i.e., $w_{\tilde{a}},u_{\tilde{a}}$) of a trapezoidal intuitionistic fuzzy number can be separately treated when arithmetic operations are done. In other words, we can divide a trapezoidal intuitionistic fuzzy number into two irrelevant sections when doing some arithmetic operations. Thus, inspired by the notion mentioned above, we propose a new and convenient method for solving the cooperative games with coalition values expressed by trapezoidal intuitionistic fuzzy numbers. In the methodology proposed in this paper, we firstly obtain the lower limit a, the upper limit c, and the most possible mean interval value $\left[b_{1},b_{2}\right]$ of players’ payoffs, which are expressed as trapezoidal intuitionistic fuzzy numbers $\tilde{a}=<(a,b_{1},b_{2},c);w_{\tilde{a}},u_{\tilde{a}}>$. Then, we construct a quadratic programming model to solve the membership degrees and nonmembership degrees of players’ payoffs.

## 3. Quadratic Programming Model for Solving Cooperative Games with Trapezoidal Intuitionistic Fuzzy Numbers

### 3.1. Cooperative Games with Coalition Values Expressed by Trapezoidal Intuitionistic Fuzzy Numbers

In this section, we discuss the mathematical form of expression of the fuzzy cooperative game $\tilde{\upsilon }$ with coalition values expressed by trapezoidal intuitionistic fuzzy numbers. Formally, a cooperative game with coalition values expressed by trapezoidal intuitionistic fuzzy numbers is an ordered pair $<N,\tilde{\upsilon }>$, where N={1,2,⋯,n} represents the set of players, and $\tilde{\upsilon }:2^{N}\to I(\mathbb{R})$ is the characteristic function, where I(ℝ) is the set of all nonempty trapezoidal intuitionistic fuzzy numbers in ℝ. The form of the coalition value of the coalition S⊆N is given as $\tilde{\upsilon }(S)=<(\upsilon^{l}(S),\upsilon^{m_{1}}(S),\upsilon^{m_{2}}(S),\upsilon^{r}(S));w_{\tilde{\upsilon }(S)},u_{\tilde{\upsilon }(S)}>$, where $\upsilon^{l}(S)$ and $\upsilon^{r}(S)$ are respectively the most pessimistic and the most optimistic predictions, and the intermediate values of $\left[\upsilon^{m_{1}}(S),\upsilon^{m_{2}}(S)\right]$ are the most possible values that coalition S could receive on its own. ∅ is a special set with no player in it, and we have $\tilde{\upsilon }(\emptyset )=0$. For the sake of convenience, we usually replace $\bar{\upsilon }(S\backslash \{i\})$, $\bar{\upsilon }(S\cup \{i\})$, $\bar{\upsilon }(\{i\})$, and $\bar{\upsilon }(\{i,j\})$ with $\bar{\upsilon }(S\backslash i)$, $\bar{\upsilon }(S\cup i)$, $\bar{\upsilon }(i)$, and $\bar{\upsilon }(i,j)$, respectively. In the sequent, we denote by ${\tilde{G}}^{N}$ the family of all cooperative games with coalition values expressed by trapezoidal intuitionistic fuzzy numbers with player set N and by $2^{N}$ the family of all subsets S⊆N.

### 3.2. The Solution Concept of Trapezoidal Intuitionistic Fuzzy Cooperative Games

For any fuzzy cooperative game $\tilde{\upsilon }\in {\tilde{G}}^{N}$ with coalition values expressed by trapezoidal intuitionistic fuzzy numbers, it is sensible that every player in coalition S⊆N should obtain a trapezoidal intuitionistic fuzzy payoff from the cooperation because the coalition values are expressed by trapezoidal intuitionistic fuzzy numbers. We denote by ${\tilde{x}}_{i}(\tilde{\upsilon })=<(x_{i}^{l}(\tilde{\upsilon }),x_{i}^{m_{1}}(\tilde{\upsilon }),x_{i}^{m_{2}}(\tilde{\upsilon }),x_{i}^{r}(\tilde{\upsilon }));w_{{\tilde{x}}_{i}},u_{{\tilde{x}}_{i}}>$ the trapezoidal intuitionistic fuzzy payoff allocated to the player i∈N when the grand coalition N is reached and by $\tilde{x}(\tilde{\upsilon })={\left({\tilde{x}}_{1}(\tilde{\upsilon }),{\tilde{x}}_{2}(\tilde{\upsilon }),\cdots ,{\tilde{x}}_{n}(\tilde{\upsilon })\right)}^{T}$ for the vector of the trapezoidal intuitionistic fuzzy payoffs for all n players in the grand coalition N. Moreover, we defined the sum of all players’ trapezoidal intuitionistic fuzzy payoffs in the coalition S as follows:

(7) $$\tilde{x}(\tilde{\upsilon },S)=\sum_{i\in S}{\tilde{x}}_{i}(\tilde{\upsilon })$$

The conclusion can be easily drawn from Equation (3) that $\tilde{x}(\tilde{\upsilon },S)$ is also a trapezoidal intuitionistic fuzzy number, which can be expressed as the following one:

(8) $$\tilde{x}(\tilde{\upsilon },S)=<(\sum_{i\in S}\left(x_{i}^{l}(\tilde{\upsilon }\right),\sum_{i\in S}x_{i}^{m_{1}}(\tilde{\upsilon }),\sum_{i\in S}x_{i}^{m_{2}}(\tilde{\upsilon }),\sum_{i\in S}x_{i}^{r}(\tilde{\upsilon }));w_{\tilde{x}(\tilde{\upsilon },S)},u_{\tilde{x}(\tilde{\upsilon },S)}>,$$

where $w_{\tilde{x}(\tilde{\upsilon },S)}=\underset{i\in S}{\min}\{w_{{\tilde{x}}_{i}}\}$, $u_{\tilde{x}(\tilde{\upsilon },S)}=\underset{i\in S}{\max}\{u_{{\tilde{x}}_{i}}\}$.

### 3.3. A Quadratic Programming Model for Solving the Numerical Value Parts Based on the Least Square Method

As stated in the aforementioned Section 2.2, the forward part (i.e., $\left(a,b_{1},b_{2},c\right)$) and the latter part (i.e., $w_{\tilde{a}},u_{\tilde{a}}$) of a trapezoidal intuitionistic fuzzy number $\tilde{a}=<(a,b_{1},b_{2},c);w_{\tilde{a}},u_{\tilde{a}}>$ do not interact when arithmetic operations are done. Therefore, we can measure the difference in the numerical value between the two trapezoidal intuitionistic fuzzy numbers $\tilde{x}(\tilde{\upsilon },S)$ and $\tilde{\upsilon }(S)$ using only their forward parts $\left(\sum_{i\in S}\left(x_{i}^{l}(\tilde{\upsilon }\right),\sum_{i\in S}x_{i}^{m_{1}}(\tilde{\upsilon }),\sum_{i\in S}x_{i}^{m_{2}}(\tilde{\upsilon }),\sum_{i\in S}x_{i}^{r}(\tilde{\upsilon })\right)$ and $\left(\upsilon^{l}(S),\upsilon^{m_{1}}(S),\upsilon^{m_{2}}(S),\upsilon^{r}(S)\right)$, which can be considered as two trapezoidal fuzzy numbers. Borrowing ideas from the concept of distance of two trapezoidal fuzzy numbers, we define the square of the distance in the numerical value between the two trapezoidal intuitionistic fuzzy numbers $\tilde{x}(\tilde{\upsilon },S)$ and $\tilde{\upsilon }(S)$ for the coalition S⊆N as follows:

(9) $$\begin{array}{ll} d(\tilde{x}(\tilde{\upsilon },S),\tilde{\upsilon }(S))= & {\left(\sum_{i\in S}\left(x_{i}^{l}(\tilde{\upsilon }\right)-\upsilon^{l}(S)\right)}^{2}+{\left(\sum_{i\in S}x_{i}^{m_{1}}(\tilde{\upsilon })-\upsilon^{m_{1}}(S)\right)}^{2}\\ & +{\left(\sum_{i\in S}x_{i}^{m_{2}}(\tilde{\upsilon })-\upsilon^{m_{2}}(S)\right)}^{2}+{\left(\sum_{i\in S}x_{i}^{r}(\tilde{\upsilon })-\upsilon^{r}(S)\right)}^{2}. \end{array}$$

Hence, the sum of the squares of the distances in the numerical value between the two trapezoidal intuitionistic fuzzy numbers $\tilde{x}(\tilde{\upsilon },S)$ and $\tilde{\upsilon }(S)$ for all coalitions S⊆N can be constructed as follows:

(10) $$\begin{array}{ll} D(\tilde{x}(\tilde{\upsilon })) & =\sum_{S\subseteq N}d(\tilde{x}(\tilde{\upsilon },S),\tilde{\upsilon }(S))\\ & \;=\sum_{S\subseteq N}[{\left(\sum_{i\in S}\left(x_{i}^{l}(\tilde{\upsilon }\right)-\upsilon^{l}(S)\right)}^{2}+{\left(\sum_{i\in S}x_{i}^{m_{1}}(\tilde{\upsilon })-\upsilon^{m_{1}}(S)\right)}^{2}\\ & \;+{\left(\sum_{i\in S}x_{i}^{m_{2}}(\tilde{\upsilon })-\upsilon^{m_{2}}(S)\right)}^{2}+{\left(\sum_{i\in S}x_{i}^{r}(\tilde{\upsilon })-\upsilon^{r}(S)\right)}^{2}]. \end{array}$$

It is obvious that the cooperative surplus is known and fixed for any cooperative game with coalition values expressed by trapezoidal intuitionistic fuzzy numbers. Consequently, if one obtains more profit from the cooperation, the others will win less. That is to say, if one pays less, the others will undertake more. However, they will accept such a profit allocation or cost sharing scheme where all the $\tilde{x}(\tilde{\upsilon },S)$ are as near to $\tilde{\upsilon }(S)$ as possible for all coalitions S⊆N, because it can embody both an impartiality principle and an effectivity principle. From the angle of all players’ payoffs, $D(\tilde{x}(\tilde{\upsilon }))$ can be considered to be a dissatisfaction function. We can obtain the forward parts (i.e., numerical value parts) of the optimal trapezoidal intuitionistic fuzzy payoff vector ${\tilde{x}}^{*}(\tilde{\upsilon })={\left({\tilde{x}}_{1}^{*}(\tilde{\upsilon }),{\tilde{x}}_{2}^{*}(\tilde{\upsilon }),\cdots ,{\tilde{x}}_{n}^{*}(\tilde{\upsilon })\right)}^{T}$ though solving the following quadratic model as follows:

$$\min\{D(\tilde{x}(\tilde{\upsilon }))\}$$

i.e.,

(11) $$\begin{array}{l} \min\{\sum_{S\subseteq N}[{\left(\sum_{i\in S}\left(x_{i}^{l}(\tilde{\upsilon }\right)-\upsilon^{l}(S)\right)}^{2}+{\left(\sum_{i\in S}x_{i}^{m_{1}}(\tilde{\upsilon })-\upsilon^{m_{1}}(S)\right)}^{2}\\ \;+{\left(\sum_{i\in S}x_{i}^{m_{2}}(\tilde{\upsilon })-\upsilon^{m_{2}}(S)\right)}^{2}+{\left(\sum_{i\in S}x_{i}^{r}(\tilde{\upsilon })-\upsilon^{r}(S)\right)}^{2}]\} \end{array}$$

### 3.4. An Improved Model Considering Efficiency and Its Optimal Solution

As stated in the aforementioned Section 3.3, we can easily obtain the numerical value parts of the optimal trapezoidal intuitionistic fuzzy payoffs of all players i∈N through solving Equation (11). However, Equation (11) has not taken into account the efficiency: $\left(\sum_{i\in N}\left(x_{i}^{l}(\tilde{\upsilon }\right),\sum_{i\in N}x_{i}^{m_{1}}(\tilde{\upsilon }),\sum_{i\in N}x_{i}^{m_{2}}(\tilde{\upsilon }),\sum_{i\in N}x_{i}^{r}(\tilde{\upsilon }))=(\upsilon^{l}(N),\upsilon^{m_{1}}(N),\upsilon^{m_{2}}(N),\upsilon^{r}(N)\right)$. It is well known that the profit allocation scheme will not be satisfactory if the efficiency has not been considered not only in classical cooperative games but also in fuzzy cooperative games. The efficiency holds a special weigh in both classical cooperative games and fuzzy cooperative games. A trapezoidal intuitionistic fuzzy payoff vector ${\tilde{x}}^{*}(\tilde{\upsilon })={\left({\tilde{x}}_{1}^{*}(\tilde{\upsilon }),{\tilde{x}}_{2}^{*}(\tilde{\upsilon }),\cdots ,{\tilde{x}}_{n}^{*}(\tilde{\upsilon })\right)}^{T}$ is said to be efficient or a preimputation if $\left(\sum_{i\in N}\left(x_{i}^{l}(\tilde{\upsilon }\right),\sum_{i\in N}x_{i}^{m_{1}}(\tilde{\upsilon }),\sum_{i\in N}x_{i}^{m_{2}}(\tilde{\upsilon }),\sum_{i\in N}x_{i}^{r}(\tilde{\upsilon })\right)$ is equal to $\left(\upsilon^{l}(N),\upsilon^{m_{1}}(N),\upsilon^{m_{2}}(N),\upsilon^{r}(N)\right)$. In the following, we mainly introduce the method for solving the quadratic programming model (12), which takes efficiency into account.

(12) $$\begin{array}{l} \min\{\sum_{S\subseteq N}[{\left(\sum_{i\in S}x_{i}^{l}(\tilde{\upsilon })-\upsilon^{l}(S)\right)}^{2}+{\left(\sum_{i\in S}x_{i}^{m_{1}}(\tilde{\upsilon })-\upsilon^{m_{1}}(S)\right)}^{2}\\ \;+{\left(\sum_{i\in S}x_{i}^{m_{2}}(\tilde{\upsilon })-\upsilon^{m_{2}}(S)\right)}^{2}+{\left(\sum_{i\in S}x_{i}^{r}(\tilde{\upsilon })-\upsilon^{r}(S)\right)}^{2}]\}\\ s.t.\left\{ \begin{array}{l} \sum_{i\in N}x_{i}^{l}(\tilde{\upsilon })=\upsilon^{l}(N)\\ \sum_{i\in N}x_{i}^{m_{1}}(\tilde{\upsilon })=\upsilon^{m_{1}}(N)\\ \sum_{i\in N}x_{i}^{m_{2}}(\tilde{\upsilon })=\upsilon^{m_{2}}(N)\\ \sum_{i\in N}x_{i}^{r}(\tilde{\upsilon })=\upsilon^{r}(N) \end{array}\right. \end{array}$$

Using the Lagrange multiplier method, Equation (12) can be rewritten as follows:

$$\min\{D(\tilde{x},\lambda ,\mu ,\delta ,\psi )\},$$

where

(13) $$\begin{array}{ll} D(\tilde{x},\lambda ,\mu ,\delta ,\psi )= & \sum_{S\subseteq N}[{\left(\sum_{i\in S}x_{i}^{l}(\tilde{\upsilon })-\upsilon^{l}(S)\right)}^{2}+{\left(\sum_{i\in S}x_{i}^{m_{1}}(\tilde{\upsilon })-\upsilon^{m_{1}}(S)\right)}^{2}+{\left(\sum_{i\in S}x_{i}^{m_{2}}(\tilde{\upsilon })-\upsilon^{m_{2}}(S)\right)}^{2}+{\left(\sum_{i\in S}x_{i}^{r}(\tilde{\upsilon })-\upsilon^{r}(S)\right)}^{2}]\\ & +\lambda (\sum_{i\in S}x_{i}^{l}(\tilde{\upsilon })-\upsilon^{l}(N))+\mu (\sum_{i\in S}x_{i}^{m_{1}}(\tilde{\upsilon })-\upsilon^{m_{1}}(N))+\delta (\sum_{i\in S}x_{i}^{m_{2}}(\tilde{\upsilon })-\upsilon^{m_{2}}(S))+\psi (\sum_{i\in S}x_{i}^{m_{2}}x_{i}^{r}(\tilde{\upsilon })-\upsilon^{r}(S)) \end{array}$$

The partial derivatives of $D(\tilde{x},\lambda ,\mu ,\delta ,\psi )$ with respect to the variables $x_{j}^{l}(\tilde{\upsilon })$, $x_{j}^{m_{1}}(\tilde{\upsilon })$, $x_{j}^{m_{2}}(\tilde{\upsilon })$, $x_{j}^{r}(\tilde{\upsilon })$ (j∈S⊆N), λ, μ, δ, and ψ are obtained as follows:

(14) $$\frac{\partial D(\tilde{x},\lambda ,\mu ,\delta ,\psi )}{\partial x_{j}^{l}(\bar{\upsilon })}=2\sum_{S\subseteq N:j\in S}\left(\sum_{i\in S}x_{i}^{l}(\tilde{\upsilon })-\upsilon^{l}(S)\right)+\lambda \;(j=1,2,\cdots ,n),$$

(15) $$\frac{\partial D(\tilde{x},\lambda ,\mu ,\delta ,\psi )}{\partial x_{j}^{m_{1}}(\bar{\upsilon })}=2\sum_{S\subseteq N:j\in S}\left(\sum_{i\in S}x_{i}^{m_{1}}(\tilde{\upsilon })-\upsilon^{m_{1}}(S)\right)+\mu \;(j=1,2,\cdots ,n),$$

(16) $$\frac{\partial D(\tilde{x},\lambda ,\mu ,\delta ,\psi )}{\partial x_{j}^{m_{2}}(\bar{\upsilon })}=2\sum_{S\subseteq N:j\in S}\left(\sum_{i\in S}x_{i}^{m_{2}}(\tilde{\upsilon })-\upsilon^{m_{2}}(S)\right)+\delta \;(j=1,2,\cdots ,n),$$

(17) $$\frac{\partial D(\tilde{x},\lambda ,\mu ,\delta ,\psi )}{\partial x_{j}^{r}(\bar{\upsilon })}=2\sum_{S\subseteq N:j\in S}\left(\sum_{i\in S}x_{i}^{r}(\tilde{\upsilon })-\upsilon^{r}(S)\right)+\psi \;(j=1,2,\cdots ,n),$$

(18) $$\frac{\partial D(\tilde{x},\lambda ,\mu ,\delta ,\psi )}{\partial \lambda }=\sum_{i=1}^{n}x_{i}^{l}(\tilde{\upsilon })-\upsilon^{l}(N),$$

(19) $$\frac{\partial D(\tilde{x},\lambda ,\mu ,\delta ,\psi )}{\partial \mu }=\sum_{i\in S}x_{i}^{m_{1}}(\tilde{\upsilon })-\upsilon^{m_{1}}(N),$$

(20) $$\frac{\partial D(\tilde{x},\lambda ,\mu ,\delta ,\psi )}{\partial \delta }=\sum_{i\in S}x_{i}^{m_{2}}(\tilde{\upsilon })-\upsilon^{m_{2}}(N)$$

and

(21) $$\frac{\partial D(\tilde{x},\lambda ,\mu ,\delta ,\psi )}{\partial \psi }=\sum_{i\in S}x_{i}^{r}(\tilde{\upsilon })-\upsilon^{r}(N),$$

respectively.

Let the partial derivatives of $D(\tilde{x},\lambda ,\mu ,\delta ,\psi )$ with respect to the variables $x_{j}^{l}(\tilde{\upsilon })$ (j∈S⊆N) and λ be equal to 0, respectively. Then, we can obtain

(22) $$2\sum_{S\subseteq N:j\in S}\left(\sum_{i\in S}x_{i}^{l*}(\tilde{\upsilon })-\upsilon^{l}(S)\right)+\lambda^{*}=0\;(j=1,2,\cdots ,n)$$

and

(23) $$\sum_{i=1}^{n}x_{i}^{l*}(\tilde{\upsilon })=\upsilon^{l}(N)$$

It easily follows from Equation (22) that

(24) $$2\times 2^{n-1}x_{i}^{l*}(\tilde{\upsilon })+2\times \sum_{j\in Ni}2^{n-2}x_{j}^{l*}(\tilde{\upsilon })-2\sum_{S:i\in S}\upsilon^{l}(S)+\lambda^{*}=0,$$

i.e.,

(25) $$2^{n-1}x_{i}^{l*}(\tilde{\upsilon })+2^{n-1}\upsilon^{l}(N)-2\sum_{S:i\in S}\upsilon^{l}(S)+\lambda^{*}=0,$$

Obviously, we have

(26) $$x_{i}^{l*}(\tilde{\upsilon })=\frac{2\sum_{S:i\in S}\upsilon^{l}(S)-2^{n-1}\upsilon^{l}(N)-\lambda^{*}}{2^{n-1}}$$

In order to obtain $x_{i}^{l*}(\tilde{\upsilon })$, it is necessary for us to solve $\lambda^{*}$ firstly. Combining Equation (23) with Equation (26), we obtain

(27) $$\sum_{i=1}^{n}\frac{2\sum_{S:i\in S}\upsilon^{l}(S)-2^{n-1}\upsilon^{l}(N)-\lambda^{*}}{2^{n-1}}=\upsilon^{l}(N).$$

Thus, we have

(28) $$\lambda^{*}=\frac{2\sum_{S\subseteq N(S\neq \emptyset )}s\upsilon^{l}(S)}{n}-2^{n-1}\upsilon^{l}(N)-\frac{2^{n-1}}{n}\upsilon^{l}(N)$$

According to Equations (26) and (28), we obtain

(29) $$\begin{array}{ll} x_{i}^{l*}(\tilde{\upsilon }) & =\frac{2\sum_{S:i\in S}\upsilon^{l}(S)-2^{n-1}\upsilon^{l}(N)-\lambda^{*}}{2^{n-1}}=\frac{2\sum_{S:i\in S}\upsilon^{l}(S)-2^{n-1}\upsilon^{l}(N)-(\frac{2\sum_{S\subseteq N(S\neq \emptyset )}s\upsilon^{l}(S)}{n}-2^{n-1}\upsilon^{l}(N)-\frac{2^{n-1}}{n}\upsilon^{l}(N))}{2^{n-1}}\\ & =\frac{2\sum_{S:i\in S}\upsilon^{l}(S)-(\frac{2\sum_{S\subseteq N(S\neq \emptyset )}s\upsilon^{l}(S)}{n}-\frac{2^{n-1}}{n}\upsilon^{l}(N))}{2^{n-1}}=\frac{\upsilon^{l}(N)}{n}+\frac{1}{n2^{n-2}}(n\sum_{S:i\in S}\upsilon^{l}(S)-\sum_{S\subseteq N(S\neq \emptyset )}s\upsilon^{l}(S)) \end{array}$$

i.e.,

(30) $$x_{i}^{l*}(\tilde{\upsilon })=\frac{\upsilon^{l}(N)}{n}+\frac{1}{n2^{n-2}}(n\sum_{S:i\in S}\upsilon^{l}(S)-\sum_{S\subseteq N(S\neq \emptyset )}s\upsilon^{l}(S))$$

In the same way, let the partial derivatives of $D(\tilde{x},\lambda ,\mu ,\delta ,\psi )$ with respect to the variables $x_{j}^{m_{1}}(\tilde{\upsilon })$, $x_{j}^{m_{2}}(\tilde{\upsilon })$, $x_{j}^{r}(\tilde{\upsilon })$ (j∈S⊆N), μ, δ, and ψ be equal to 0, respectively, and we can obtain the following results through some mathematical derivations:

(31) $${x_{i}^{m_{1}}}^{*}(\tilde{\upsilon })=\frac{\upsilon^{m_{1}}(N)}{n}+\frac{1}{n2^{n-2}}(n\sum_{S:i\in S}\upsilon^{m_{1}}(S)-\sum_{S\subseteq N(S\neq \emptyset )}s\upsilon^{m_{1}}(S))$$

(32) $${x_{i}^{m_{2}}}^{*}(\tilde{\upsilon })=\frac{\upsilon^{m_{2}}(N)}{n}+\frac{1}{n2^{n-2}}(n\sum_{S:i\in S}\upsilon^{m_{2}}(S)-\sum_{S\subseteq N(S\neq \emptyset )}s\upsilon^{m_{2}}(S))$$

and

(33) $$x_{i}^{r*}(\tilde{\upsilon })=\frac{\upsilon^{r}(N)}{n}+\frac{1}{n2^{n-2}}(n\sum_{S:i\in S}\upsilon^{r}(S)-\sum_{S\subseteq N(S\neq \emptyset )}s\upsilon^{r}(S))$$

So far we have obtained the numerical value parts of the optimal trapezoidal intuitionistic fuzzy payoff vector ${\tilde{x}}^{*}(\tilde{\upsilon })={\left({\tilde{x}}_{1}^{*}(\tilde{\upsilon }),{\tilde{x}}_{2}^{*}(\tilde{\upsilon }),\cdots ,{\tilde{x}}_{n}^{*}(\tilde{\upsilon })\right)}^{T}$ (i.e., $x_{i}^{l*}(\tilde{\upsilon })$, ${x_{i}^{m_{1}}}^{*}(\tilde{\upsilon })$, ${x_{i}^{m_{2}}}^{*}(\tilde{\upsilon })$, and $x_{i}^{r*}(\tilde{\upsilon })$). In the following, we will focus on how to obtain the latter parts (i.e., the membership degrees and nonmembership degrees) of the optimal trapezoidal intuitionistic fuzzy payoff vector ${\tilde{x}}^{*}(\tilde{\upsilon })$.

### 3.5. A Quadratic Programming Model for Solving the Membership Degrees and Nonmembership Degrees of the Optimal Solution

As stated earlier, the players will accept the profit allocation or cost sharing scheme where all the $\tilde{x}(\tilde{\upsilon },S)$ are as near to $\tilde{\upsilon }(S)$ as possible for all coalitions S⊆N. Based on the above view and the arithmetical operations of trapezoidal intuitionistic fuzzy numbers, to solve the latter parts (i.e., the membership degrees and nonmembership degrees) of the optimal trapezoidal intuitionistic fuzzy payoff vector ${\tilde{x}}^{*}(\tilde{\upsilon })={\left({\tilde{x}}_{1}^{*}(\tilde{\upsilon }),{\tilde{x}}_{2}^{*}(\tilde{\upsilon }),\cdots ,{\tilde{x}}_{n}^{*}(\tilde{\upsilon })\right)}^{T}$, a quadratic programming model is constructed as follows:

(34) $$\begin{array}{l} \min\{\sum_{i\in N}[{\left(w_{{\tilde{x}}_{i}}-w_{\tilde{\upsilon }(i)}\right)}^{2}+{\left(u_{{\tilde{x}}_{i}}-u_{\tilde{\upsilon }(i)}\right)}^{2}]\}\\ s.t.\left\{ \begin{array}{l} \underset{i\in N}{\min}\{w_{{\tilde{x}}_{i}}\}=w_{\tilde{\upsilon }(N)}\\ \underset{i\in N}{\max}\{u_{{\tilde{x}}_{i}}\}=u_{\tilde{\upsilon }(N)} \end{array}\right. \end{array}$$

Equation (34) can be quickly solved by Lingo software.

## 4. An Example Demonstrating the Cost-Sharing of Ecological Construction in Fujian Province, China

Along with the rapid development of the economy and social productivity, the material progress of human beings has been promoted enormously. However, owing to the excessive consumption of resources, the ecological environment that humans live upon is suffering severe damage and increasingly facing threats. Therefore, it is imperative for governments around the world that prompt and effective technological measures be taken to improve ecological environment for the sake of future generations. However, the theory and practice of ecological construction involves many different aspects and interested parties (i.e., players). Owing to the fact that the regional characteristics of ecological construction is obvious and remarkable, the ecological chains among one country or region interact intimately and cannot be separated, and the ecological environment of one region has great influence on adjacent areas; as such, it is difficult and almost impossible for one country or organization to carry out ecological construction and environmental protection successfully on its own. That is to say, the advancement of ecological construction needs regional linkage and cooperation. Generally speaking, the construction of ecological civilization needs a long time, involves many interested parties, and contains much uncertainty and fuzziness. There actually exist both competition and cooperation in the process of ecological construction. Ecological construction involves multiple stakeholders, who not only have a common and magnificent goal, but also pursue the maximization of their self-interests. Ecological construction is a typical type of fuzzy cooperative game.

Take Fujian province in China for instance. Min River is the largest river in Fujian province, of which the ecosystem health of the upstream district plays a prime important role in the sustainable development of the society and economy of Fujian province. However, owing to the overexploitation of minerals, the excessive construction of hydropower stations, and the excessive emission of animal dung, the ecosystem degradation of Min River is increasingly severe. It is time that an ecological and environment warning system should be established to forecast the ecological degradation and the deterioration of environmental quality caused by human activities. Moreover, some urgent measures must be taken to improve ecological quality. It is well known that Min River flows through three main cities in Fujian province, which are Fuzhou city, Sanming city, and Nanping city, respectively. In the following, for the sake of convenience, we replace Fuzhou city, Sanming city, and Nanping city with player 1, player 2, and player 3, respectively. Because of the long cycle of construction, the substantial input of funds, and the fuzziness and uncertainty existing in the process of ecological construction, it is almost impossible to estimate precisely the construction cost. That is to say, it is difficult to express the cost of ecological construction by real numbers. Fortunately, the trapezoidal intuitionistic fuzzy numbers can suitably deal with the information of fuzzy uncertainty. Through the feasibility analysis and cost estimate, if the three cities determine to take measures to improve the ecological environment of Min River all alone, the costs of ecological construction can be expressed by trapezoidal intuitionistic fuzzy numbers as follows: $\tilde{\upsilon }(1)=<(80,100,120,150);0.6,0.3>$, $\tilde{\upsilon }(2)=<(150,180,250,300);0.4,0.2>$, and $\tilde{\upsilon }(3)=<(400,600,800,850);0.7,0.1>$. The cost of ecological construction to player 1 (i.e., $\tilde{\upsilon }(1)=<(80,100,120,150);0.6,0.3>$) represent a possible range, which is approximately equal to the interval [100,120]. In other words, the most possible value is any intermediate value *x* between 100 and 120 with the membership degree 0.6 and nonmembership degree 0.3; the optimistic value is 80 with the membership degree 0 and nonmembership degree 1; the pessimistic value is 150 with the membership degree 0 and nonmembership degree 1. The meanings of the costs of ecological construction to player 2 and player 3 are completely the same as player 1. However, if they decide to cooperate with each other, the costs of ecological construction can be reduced to a great extent. For example, if player 1 and play 2 form a coalition to improve the ecological environment of Min River together, the sum of the costs can be expressed by $\tilde{\upsilon }(1,2)=<(180,220,300,380);0.5,0.4>$. Similarly, other possible coalitions’ values can be also expressed by trapezoidal intuitionistic fuzzy numbers as follows: $\tilde{\upsilon }(1,3)=<(350,500,700,850);0.4,0.3>$, $\tilde{\upsilon }(2,3)=<(380,580,820,1000);0.6,0.2>$, and

$\tilde{\upsilon }(1,2,3)=<(400,650,900,1100);0.5,0.2>$, respectively.

According to Equation (30), we have

(35) $$\begin{array}{ll} x_{1}^{l*}(\tilde{\upsilon }) & =\frac{\upsilon^{l}(N)}{n}+\frac{1}{n2^{n-2}}(n\sum_{S:i\in S}\upsilon^{l}(S)-\sum_{S\subseteq N(S\neq \emptyset )}s\upsilon^{l}(S))\\ & =\frac{400}{3}+\frac{1}{6}[3\times (80+180+350+400)-(80+150+400+2\times 180+2\times 350+2\times 380+3\times 400)]\\ & =30 \end{array}$$

i.e., $x_{1}^{l*}(\tilde{\upsilon })=30$.

In the same way, we can easily obtain the following results: $x_{2}^{l*}(\tilde{\upsilon })=80$, and $x_{3}^{l*}(\tilde{\upsilon })=290$.

Similarly, by using Equations (31)–(33), we obtain

${x_{1}^{m_{1}}}^{*}(\tilde{\upsilon })=46.7$, ${x_{2}^{m_{1}}}^{*}(\tilde{\upsilon })=126.7$, ${x_{3}^{m_{1}}}^{*}(\tilde{\upsilon })=476.7$, ${x_{1}^{m_{2}}}^{*}(\tilde{\upsilon })=58.3$, ${x_{2}^{m_{2}}}^{*}(\tilde{\upsilon })=183.3$, ${x_{3}^{m_{2}}}^{*}(\tilde{\upsilon })=658.3$, $x_{1}^{r*}(\tilde{\upsilon })=96.7$, $x_{2}^{r*}(\tilde{\upsilon })=246.7$, and $x_{3}^{r*}(\tilde{\upsilon })=756.7$, respectively.

So far, we have got the numerical value parts of the optimal trapezoidal intuitionistic fuzzy payoff vector ${\tilde{x}}^{*}(\tilde{\upsilon })$, which are (30,46.7,58.3,96.7), (80,126.7,183.3,246.7), and (290,476.7,658.3,756.7), respectively. In the following, we focus on solving the membership degrees and nonmembership degrees of the optimal trapezoidal intuitionistic fuzzy payoff vector ${\tilde{x}}^{*}(\tilde{\upsilon })$.

According to Equation (34), the following procedures are written on Lingo software:

(36) $$\begin{array}{l} \min={\left(w_{{\tilde{x}}_{1}}-0.6\right)}^{2}+{\left(u_{{\tilde{x}}_{1}}-0.3\right)}^{2}+{\left(w_{{\tilde{x}}_{2}}-0.4\right)}^{2}+{\left(u_{{\tilde{x}}_{2}}-0.2\right)}^{2}+{\left(w_{\tilde{x}3}-0.7\right)}^{2}+{\left(u_{{\tilde{x}}_{3}}-0.1\right)}^{2}\\ s.t.\left\{ \begin{array}{l} \min\{w_{{\tilde{x}}_{1}},w_{{\tilde{x}}_{2}},w_{{\tilde{x}}_{3}}\}=0.5\\ \max\{u_{{\tilde{x}}_{1}},u_{{\tilde{x}}_{2}},u_{{\tilde{x}}_{3}}\}=0.2 \end{array}\right. \end{array}$$

Then, we can obtain the membership degrees and nonmembership degrees of ${\tilde{x}}^{*}(\tilde{\upsilon })$ as follows: $w_{{\tilde{x}}_{1}}=0.6$, $w_{{\tilde{x}}_{2}}=0.5$, $w_{{\tilde{x}}_{3}}=0.7$, $u_{{\tilde{x}}_{1}}=0.2$, $u_{{\tilde{x}}_{2}}=0.2$, and $u_{{\tilde{x}}_{3}}=0.1$, respectively.

Consequently, we have obtained the optimal trapezoidal intuitionistic fuzzy payoff vector ${\tilde{x}}^{*}(\tilde{\upsilon })={\left({\tilde{x}}_{1}^{*}(\tilde{\upsilon }),{\tilde{x}}_{2}^{*}(\tilde{\upsilon }),\cdots ,{\tilde{x}}_{n}^{*}(\tilde{\upsilon })\right)}^{T}$, which are expressed as follows:

$${\tilde{x}}_{1}^{*}(\tilde{\upsilon })=<(x_{1}^{l*}(\tilde{\upsilon }),x_{1}^{m_{1}*}(\tilde{\upsilon }),x_{1}^{m_{2}*}(\tilde{\upsilon }),x_{1}^{r*}(\tilde{\upsilon }));w_{{\tilde{x}}_{1}},u_{{\tilde{x}}_{1}}>=<(30,46.7,58.3,96.7),0.6,0.2>,$$

$${\tilde{x}}_{2}^{*}(\tilde{\upsilon })=<(x_{2}^{l*}(\tilde{\upsilon }),x_{2}^{m_{1}*}(\tilde{\upsilon }),x_{2}^{m_{2}*}(\tilde{\upsilon }),x_{2}^{r*}(\tilde{\upsilon }));w_{{\tilde{x}}_{2}},u_{{\tilde{x}}_{2}}>=<(80,126.7,183.3,246.7),0.5,0.2>$$

and

$${\tilde{x}}_{3}^{*}(\tilde{\upsilon })=<(x_{3}^{l*}(\tilde{\upsilon }),x_{3}^{m_{1}*}(\tilde{\upsilon }),x_{3}^{m_{2}*}(\tilde{\upsilon }),x_{3}^{r*}(\tilde{\upsilon }));w_{{\tilde{x}}_{3}},u_{{\tilde{x}}_{3}}>=<(290,476.7,658.3,756.7),0.7,0.1>.$$

## 5. Discussion

According to the derivation process of the formulas and the calculation methods and results, the following conclusions and advantages can easily be made:

(1) Modeling. In this paper, we constructed a quadratic programming model to solve the cooperative game with coalition values expressed by trapezoidal intuitionistic fuzzy numbers. It is an expansion of the least square prenucleolus solution concept [31]. The quadratic programming models and methods proposed in this paper always assure that the solutions are positive if all of the coalitions’ values are positive trapezoidal intuitionistic fuzzy numbers.
(2) Calculation complexity. According to the method proposed in this paper, we can easily and quickly obtain all players’ optimal trapezoidal intuitionistic fuzzy payoffs using Equations (30)–(33).
(3) Efficiency. The quadratic programming model proposed in this paper takes into account efficiency, so the allocation scheme is fairly satisfactory for all players. That is to say, the cooperative surplus is distributed thoroughly among the players. In Section 4, it can easily be seen that $\sum_{i=1}^{3}{\tilde{x}}_{i}^{*}(\tilde{\upsilon })=\tilde{\upsilon }(N)$, (i.e., 30 + 80 + 290 = 400, 46.7 + 126.7 + 476.7 = 650, 58.3 + 183.3 + 658.3 = 900, 96.7 + 246.7 + 756.7 = 1100) which implies that the cost allocation scheme satisfies the efficiency as expected. The optimal trapezoidal intuitionistic fuzzy payoff vector ${\tilde{x}}^{*}(\tilde{\upsilon })$, which is obtained through Equations (30)–(33), is said to be efficient or a preimputation.
(4) Advantages. There exists some information distortion when doing subtraction of trapezoidal intuitionistic fuzzy numbers. In this paper, we construct the optimal mathematical model based on the square of the distance in the numerical value between two trapezoidal intuitionistic fuzzy numbers, which can effectively avoid the distortion of information and enlargement of fuzziness and uncertainty brought about by subtraction of trapezoidal intuitionistic fuzzy numbers.

## 6. Conclusions

Fuzzy cooperative games can provide an effective allocation scheme for decision problems that exist which are complicated by impreciseness and uncertainty. Among the different types of fuzzy cooperative games, the one with coalition values expressed by trapezoidal intuitionistic fuzzy numbers has generated less discussion because distortion of information and enlargement of fuzziness always occurs when some arithmetical operations are done (especially subtraction). In this paper, we propose a quadratic programming model to obtain the optimal trapezoidal intuitionistic fuzzy payoffs for players when they form a coalition in order to work together to improve the ecological environment of Min River. The proposed model and method in this paper can be applied to many other areas such as the economy, management, politics, and diplomacy.

## References

[1] Estévez-Fernández A. (2008). A game theoretical approach to sharing penalties and rewards in projects. Eur. J. Oper. Res..

[2] Chevalier-Roignant B., Flath C.M., Huchzermeier A., Trigeorgis L. (2011). Strategic investment under uncertainty: A synthesis. Eur. J. Oper. Res..

[3] Seyedesfahani M.M., Biazaran M., Gharakhani M. (2011). A game theoretic approach to coordinate pricing and vertical co-op advertising in manufacturer-retailer supply chains. Eur. J. Oper. Res..

[4] Jiménez-Losada A., Fernández J.R., Ordóñez M., Grabisch M. (2010). Games on fuzzy communication structures with Choquet players. Eur. J. Oper. Res..

[5] Leng M., Zhu A. (2009). Side-payment contracts in two-person nonzero-sum supply chain games: Review, discussion and applications. Eur. J. Oper. Res..

[6] Nishizaki I., Sakawa M. (2000). Equilibrium solutions in multiobjective bimatrix games with fuzzy payoffs and fuzzy goals. Fuzzy Sets Syst..

[7] Viscolani B. (2012). Pure-strategy Nash equilibria in an advertising game with interference. Eur. J. Oper. Res..

[8] Collins W.D., Hu C. (2008). Studying interval valued matrix games with fuzzy logic. Soft Comput..

[9] Li D.F. (2011). Linear programming approach to solve interval-valued matrix games. Omega.

[10] Li D.F., Nan J.X., Zhang M.J. (2012). Interval programming models for matrix games with interval payoffs. Optim. Methods Softw..

[11] Liu S.T., Kao C. (2009). Matrix games with interval data. Comput. Ind. Eng..

[12] Avşar Z.M., Baykal-Gürsoy M. (2006). A note on two-person zero-sum communicating stochastic games. Oper. Res. Lett..

[13] Lakshmana G.N.V., Jeevaraj S., Dhanasekaran P. (2016). A linear ordering on the class of trapezoidal intuitionistic fuzzy numbers. Expert Syst. Appl..

[14] Nehi H.M. (2010). A New Ranking Method for Intuitionistic Fuzzy Numbers. Int. J. Fuzzy Syst..

[15] Garg H. (2016). A novel approach for analyzing the reliability of series-parallel system using credibility theory and different types of intuitionistic fuzzy numbers. J. Braz. Soc. Mech. Sci. Eng..

[16] Wan S.P., Wang F., Lin L.L., Dong J.Y. (2016). Some new generalized aggregation operators for triangular intuitionistic fuzzy numbers and application to multi-attribute group decision making. Comput. Ind. Eng..

[17] Li D.F. (2010). A ratio ranking method of triangular intuitionistic fuzzy numbers and its application to MADM problems. Comput. Math. Appl..

[18] Greer K., Cameron L. (2015). The use and abuse of ecological constructs. Geoforum.

[19] Mangone G. (2016). Constructing hybrid infrastructure: Exploring the potential ecological, social, and economic benefits of integrating municipal infrastructure into constructed environments. Cities.

[20] Zhao J., Liu X., Dong R., Shao G. (2015). Landsenses ecology and ecological planning toward sustainable development. Int. J. Sustain. Dev. World Ecol..

[21] Zheng R., Zhang T., Liu Z., Wang H. (2015). An EIoT system designed for ecological and environmental management of the Xianghe Segment of China’s Grand Canal. Int. J. Sustain. Dev. World Ecol..

[22] Liou Y.A. (2016). Zoning eco-environmental vulnerability for environmental management and protection. Ecol. Ind..

[23] Yu B., Xu L., Yang Z. (2015). Ecological compensation for inundated habitats in hydropower developments based on carbon stock balance. J. Clean. Prod..

[24] Guan X., Liu W., Chen M. (2016). Study on the ecological compensation standard for river basin water environment based on total pollutants control. Ecol. Ind..

[25] Reid J., Bruner A., Chow J., Malky A., Rubio J.C., Vallejos C. (2015). Ecological Compensation to Address Environmental Externalities: Lessons from South American Case Studies. J. Sustain. Forest..

[26] Eric M., Anne-Sophie D., Grémillet D., Gauthier-Clerc M., Béchet A. (2014). Combining correlative and mechanistic habitat suitability models to improve ecological compensation. Biol. Rev. Camb. Philos. Soc..

[27] Rao H., Lin C., Kong H., Jin D., Peng B. (2014). Ecological damage compensation for coastal sea area uses. Ecol. Ind..

[28] Villarroya A., Persson J., Puig J. (2014). Ecological compensation: From general guidance and expertise to specific proposals for road developments. Environ. Impact Assess. Rev..

[29] Li D.F. (2014). Decision and Game Theory in Management with Intuitionistic Fuzzy Sets.

[30] Wan S.P., Dong J.Y. (2015). Power geometric operators of trapezoidal intuitionistic fuzzy numbers and application to multi-attribute group decision making. Appl. Soft Comput..

[31] Ruiz L.M., Valenciano F., Zarzuelo J.M. (1996). The least square prenucleolus and the least square nucleolus. Two values for TU games based on the excess vector. Int. J. Games Theory.

